# Elucidating the Fundamental Process of Methyl-(5hydroxymethyl) Furan-2-Carboxylate Toxin Biosynthesis in *Curvularia lunata* Causing Maize Leaf Spot

**DOI:** 10.3390/jof10100688

**Published:** 2024-09-30

**Authors:** Zhixiang Lu, Bo Lang, Shaoqing Wang, Hongyi Liu, Xinhua Wang, Jie Chen

**Affiliations:** 1 School of Agriculture and Biology, Shanghai Jiaotong University, Shanghai 200240, China; luzhixiang@sjtu.edu.cn (Z.L.); w1214900346@163.com (S.W.);; 2 Guangxi Academy of Sciences, Nanning 530007, China; 3 State Key Laboratory of Microbial Metabolism, Shanghai Jiaotong Universtiy, Shanghai 200240, China

**Keywords:** *Curvularia lunata*, methyl-(5hydroxymethyl) furan-2-carboxylate toxin, biosynthesis, *Cladh6*

## Abstract

Maize leaf spot, which is caused by *Curvularia lunata* (Wakkre) Boedijn, was epidemic in the maize-growing regions of northeastern and northern China in the mid-1990s, where it led to large yield losses. Since then, the epidemic has evolved into a kind of common disease. In recent years, however, a tendency of becoming an epidemic disease again has been observed in some areas in China due to significant changes in climate, farming, systems and crop varieties. The significance of methyl-(5hydroxymethyl) furan-2-carboxylate (M5HF2C) as a nonspecific host toxin in causing maize leaf spot disease has been demonstrated in previous research. However, the key enzymes involved in M5HF2C toxin synthesis remain unclear. In our study, we demonstrate that the synthesis of M5HF2C toxin starts from a precursor substrate in the pathogen, furfural, which is then catalytically dehydrogenated into furoic acid via an alcohol dehydrogenase (CLADH6). The furoic acid was further confirmed as one of the raw materials for the biosynthesis of M5HF2C toxin based on deletion mutants of the alcohol dehydrogenase gene (*Cladh6*) in *C*. *lunata*, which had reduced M5HF2C toxin-producing ability; however, this ability could be restored in all deletion mutants through complementation with furoic acid, thereby confirming that furoic acid is an intermediate in the biosynthesis of M5HF2C toxin. In summary, the biosynthesis process of M5HF2C toxin in *C*. *lunata* involves three transformation steps: (1) from xylose to furfural; (2) then from furfural to furoic acid; and (3) eventually from furoic acid to M5HF2C toxin. Our research findings provide new clues in elucidating the major steps in the process of M5HF2C toxin biosynthesis in *C*. *lunata*.

## 1. Introduction

Curvularia leaf spot (CLS) is one of various diseases that occurs worldwide, including in countries in Europe, Africa, Asia, and the Americas. This disease is caused by *Curvularia lunata* (Wakker) Boedijn, whose sexual form was identified as belonging to *Cochliobolus lunata* Nelson & Haasis of the Ascomycota [1]. Since 1980, the disease has become the third-most-serious leaf disease, being found widely distributed throughout the maize-planting regions of China, including in the provinces of Jilin, Liaoning, Hebei, Henan, Shanxi and Shandong, as well as the municipalities of Beijing and Tianjin. Currently, an epidemic has been prevented by planting resistant cultivars in large areas over the past few decades. However, investigations have shown that in recent years, the incidence of disease is escalating in some regions of China; therefore, the potential risk to maize safety production posed by this disease remains a concern [2].

**Figure 1:**
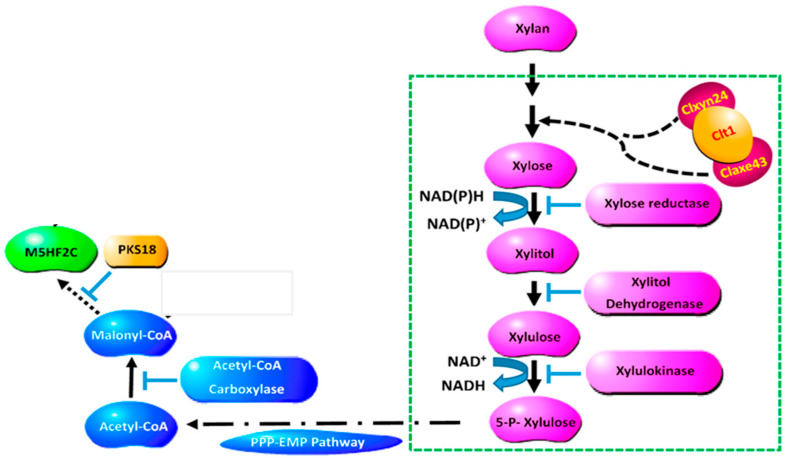
Hypothetical model of CLXYN24, CLAXE43, and CLPKS18 involvement in M5HF2C toxin biosynthesis in *C. lunata* [4]. Note: In this study, it was deduced that the synthesis of M5HF2C toxin originates from xylan, which is gradually degraded to xylose and undergoes a series of reactions such as reductive dehydrogenation to produce xylulose, which is then converted to acetyl coenzyme A via the PPP-EMP pathway. M5HF2C toxin is ultimately synthesized by a series of enzymes, including PKS18 from acetyl coenzyme A.

**Figure 2:**
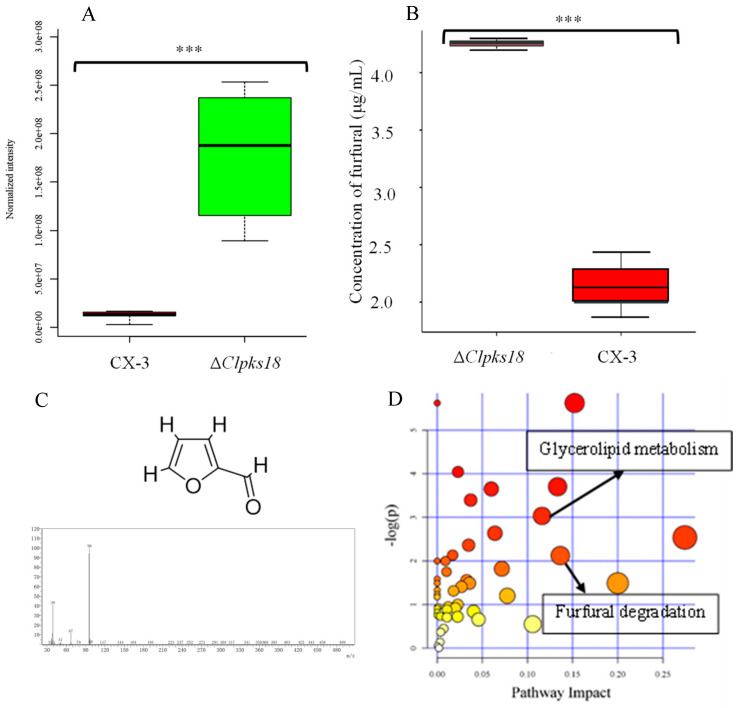
The role of furfural in M5HF2C toxin biosynthesis. (**A**) Relative quantification of furfural in the fermentation broth of each strain using metabonomics. (**B**) Absolute quantification of furfural in the fermentation broth of each strain using GC-MS. (**C**) Structure and GC-MS spectrum of furfural. (**D**) Rich factor map for KEGG enrichment analysis [6]. Note: KEGG enrichment analysis reveals significant enrichment of a number of genes related to furfural metabolism. Three asterisks (***) indicate significant differences at the 0.001 level.

**Figure 3:**
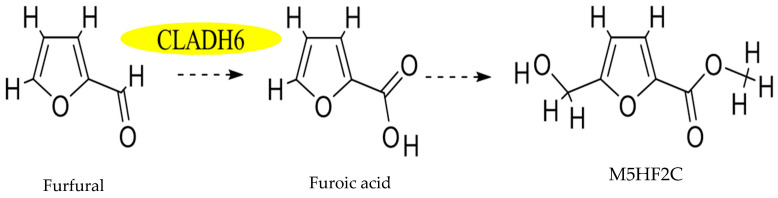
Hypothetical diagram of M5HF2C toxin biosynthesis. Note: In this study, it is deduced that furfural is a node in the synthesis of M5HF2C toxin.

In previous research, methyl-5-(hydroxymethyl)-furan-2-carboxylate was identified as a nonspecific toxin that can cause heavy electrolyte leakage from host cell membranes, impacting chlorophyll synthesis in the host leaf and eventually leading to cell death [3]. It was also demonstrated in previous research that xylanase and acetyl xylan esterase play integral roles in the biosynthesis of M5HF2C toxins; consequently, these two enzymes are also closely linked to the pathogenicity of *C*. *lunata*. In culture conditions where xylan serves as the exclusive source of carbon, mutations in xylan metabolism-related genes (Δ*clxyn24*, Δ*claxe43*, and Δ*clxyn24*&*claxe43*) resulted in a substantially reduced capacity for the production of M5HF2C toxin. However, partial restoration of the toxin production capability of these mutant strains was observed when xylose was employed as a substitute for xylan. This indicates that xylan degradation may serve in providing a source of acetyl-CoA substrate that is essential for the biosynthesis of M5HF2C toxin [4] (Figure 1). In this study, it is hypothesized that xylose is converted to acetyl coenzyme A via the PPP-EMP pathway. Also, common knowledge shows that xylose is converted into pyruvate through the glycolysis process, and then the pyruvate enters mitochondria for further conversion into acetyl CoA through the action of the pyruvate dehydrogenase complex; therefore, it is confirmed that acetyl coenzyme A is a precursor for M5HF2C toxin synthesis in some ways. However, according to the literature, simple non-enzymatic reactions can convert xylose into furfural. For example, furfural is commercially produced through the acid-catalyzed transformation of xylose present in biomass [5]. Therefore, we hypothesize that xylose produced by *C*. *lunata* degrading host plant cell walls during the course of pathogen infection in the host plant may directly serve as one of the sources of furfural. The Δ*Clpks18* strain is defective in the production of M5HF2C toxin, with levels much lower than for the wild-type strain CX-3. Through comparative secondary metabolome analysis, it was found that large amounts of furfural accumulate in the fermentation broth of Δ*Clpks18* (a single-deletion mutant of the polyketide synthase gene family) (Figure 2A). Absolute quantification using GC-MS showed that there was significantly higher furfural in the fermentation broth of Δ*Clpks18* than of the wild-type strain CX-3 (Figure 2B). These differences in extracellular secretion between the CX-3 and Δ*Clpks18* strains were shown to be significantly related to the furfural degradation metabolic pathway via KEGG pathway enrichment analysis (Figure 2D) [6]. It was recently reported that ethanol dehydrogenase demonstrates strong activity in oxidizing furfural to furoic acid [7]. A gene encoding alcohol dehydrogenase is present in the genome of the CX-3 strain, namely *Cladh6*. The levels of secreted products in Fries 3 medium indicate there is significantly higher expression in the CX-3 strain than in the mutant Δ*Clpks18* strain with defective M5HF2C toxin synthesis [6]. Thus, it is postulated that CLADH6 has the activity to oxidize furfural to furoic acid. There is speculation that furoic acid is an important intermediate product for the biosynthesis of toxin M5HF2C; in this case, furfural could be a precursor for the formation of furoic acid (Figure 3). Therefore, our research aims to provide support for these suggestions related to the potential pathway of M5HF2C toxin synthesis by using the precursors xylose, furfural, and furoic acid.

## 2. Materials and Methods

### 2.1. Materials

The Δ*clxyn24*, Δ*claxe43,* and CX-3 strains of *C*. *lunata* are kept in the plant pathology laboratory of Shanghai Jiao Tong University School of Agriculture and Biology. The pET-32a pCAMBIA1300qh vectors are deposited in the same institution.

*Escherichia coli* BL21(DE3) competent cells were purchased from TIANGEN Biotech Co., Ltd. (CB105) (Beijing, China).

*Agrobacterium tumefaciens* AGL1 competent cells were produced by our laboratory.

### 2.2. Methods

#### 2.2.1. Detection of Expression Levels of *Cladh6* in Δ*clxyn24*, Δ*claxe43* and CX-3 Strains

The Δ*clxyn24*, Δ*claxe43*, and CX-3 strains were cultured in Fries 3 medium for 7 days, their RNA was extracted and reverse transcribed into cDNA, and the expression of *Cladh6* was detected using real-time fluorescence quantitative PCR. The primers used are as follows: Cladh6F-GGGTCCAACGAACTGGGAAT/Cladh6R-TGGAAGAATACACGCTCGCC.

#### 2.2.2. Detection of Furoic Acid Content in Δ*clxyn24*, Δ*claxe43*, and CX-3 Strains

The CX-3, Δ*clxyn24*, and Δ*claxe43* strains were cultured in Fries 3 medium for 7 days, and the furoic acid content in each strain was detected using GCMS-TQ8050 NX (Shimadzu, Kyoto, Japan). Fermentation broth (50 mL) of each strain was dried in a vacuum under low-temperature (−80 °C) conditions and then redissolved in 5 mL of methanol for subsequent GC-MS testing. The conditions for GC-MS were as follows:

GC conditions: DB-WAX column (30 m × 250 µm × 0.25 µm); temperature program: 50 °C for 3 min, 10 °C/min to 130 °C, 0 min, 40 °C/min to 240 °C, 4 min, equilibrium time 0.5 min, maximum temperature 250 °C. Carrier gas (He) flow rate: 1.0 mL/min, pressure: 8.3852 psi, injection volume: 1 µL, split ratio: 5:1, split flow: 5 mL/min.

MS conditions: electron bombardment ion source; electron energy: 70 eV; transmission line temperature: 280 °C; ion source temperature: 260 °C; quantitative ion *m*/*z*: 112.00; residence time: 564.72 s; quadrupole temperature: 150 °C; activation voltage: 1.5 V; mass scanning range *m*/*z*: 30–400.

#### 2.2.3. Xylose Anaplerosis Experiment

Given the lack of enzymes in the Δ*clxyn24* and Δ*claxe43* mutants capable of converting xylan to xylose, xylose was used as the sole carbon source in cultures of Δ*clxyn24*, Δ*claxe43*, and CX-3 in Fries 3 medium for the anaplerotic experiment. Then, the production of furoic acid and M5HF2C toxin in the above three strains was characterized via detection. The method for furoic acid detection is described in Section 2.2.2. The method for M5HF2C toxin detection is described as below.

Each of the above mutant strains and wild-type strain CX-3 were cultured in Fries 3 medium for 15 days, and the production of M5HF2C toxin was detected. Fermentation broth (50 m) of each strain was freeze-dried under vacuum, reconstituted in methanol, and the M5HF2C contents were detected using 30A/Sciex Quadrupole 5500 UHPLC-MS (AB Sciex, Framingham, MA, USA).

The conditions for liquid–quantity coupling are as follows:

(1) Liquid phase conditions:

Column: ACQUITY UPLC BEHC18 2.1 × 100 mm 1.7 µm;

Column temperature: 40 °C;

Mobile phase: A—0.1% FA ACN; B—0.1% FA H_2_O.

The elution gradient of the mobile phase is detailed in Table 1.

(2) Mass spectrometry conditions

ESI negative ion mode. Air curtain gas: 35 psi; ionization voltage: 5500 V; desolventization temperature: 500 °C; atomization gas: 55 psi; auxiliary heating gas: 55 psi. The samples were analyzed using multiple reaction monitoring (MRM), and the mass spectrometry parameters of the target components are shown in Table 2.

#### 2.2.4. Construction of *Cladh6* Prokaryotic Expression System

The genome of the CX-3 strain was analyzed to obtain the full-length DNA sequence of the *Cladh6* gene, which is 1086 bp in length, encoding a total of 361 amino acids. The *Clad6* cloning primers were designed based on the CX-3 genome sequence as follows: Cladh6F + Ecol I-GCTGATATCGGATCCGAATTCATGTCTCTCCCATCAACCTT/Cladh6R + Hind III-CTCGAGTGCGGCCGCAAGCTTCTAGTTTGTTTCGTGTGGACG.

The CX-3 strain was cultured in Fries 3 medium for 5 days, and the total RNA of the hyphae was extracted. Total RNA was used as a template and reverse transcribed into single-stranded cDNA. *Cladh6* was amplified by PCR using cDNA as template.

The PCR product of the above *Cladh6* gene was purified, connected to the pET-32a vector linearized through enzyme digestion using the In-Fusion^®^ HD Cloning Kit, and then transferred into competent *E. coli* cells of the DH-5α strain, with positive clones selected from the LA resistance screening medium with added Amp (100 µg/mL), and the correct clones as verified by colony PCR were sent to Beijing Tsingke Biotech Co., Ltd., (Beijing, China) for sequencing verification. The correct plasmid, as verified through sequencing, was named pET-32a-*Cladh6*.

The TIANprep Mini Plasmid Kit (TIANGEN BIOTECH, Beijing, China) was used to extract the pET-32a-*Cladh6* vector from the correctly sequenced DH-5α strain, transfer the pET-32a-*Cladh6* vector into *E. coli* BL21(DE3) competent cells, and select positive clones from the LA resistance screening plate containing Amp (100 µg/mL). Colony PCR amplification of the *Cladh6* fragment was used for verifying whether the positive clone was correct. The strains verified using PCR were transferred to LB medium, and IPTG was added for induction. The total protein was extracted, and the expression of CLADH6 protein was detected by Western blot (expected protein size: 38.28 KDa).

#### 2.2.5. Determination of ClADH6 Activity

BL21(DE3) carrying the recombinant vector pET30a-*cladh6* was amplified and cultured at a ratio of 1:100 for 18 h, then further by shaking culture at 200 rpm and 37 °C for about 3 h until OD_600_ = 0.4–0.6. Then, 5 mL of each culture medium was mixed with 1 mM IPTG and subjected to shaking at 200 rpm and 28 °C. After 12 h, the induction culture medium was centrifuged at 10,000× *g* for 1 min to collect the bacterial cells. After rinsing once with PBS (pH 7.4), the bacterial cell pellet was suspended in 500 µL PBS (pH 7.4). The pH of the total protein solution after ultrasonic cleavage of the above BL21(DE3) was adjusted to about 6.5 using phosphate buffer. The substrate furfural was then added to the bacterial cell pellet and vortexed until the final concentration reached 70 µg/mL. At 0 h, a portion of the solution was extracted for the detection of furfural and furoic acid. After incubating at 30 °C for 24 h, the contents of furfural and furoic acid in the crude enzyme solution were again determined. The furfural and furoic acid contents in this reaction system were detected using GC-MS (Shimadzu GCMS-TQ8050 NX) following the method in Section 2.2.2. Furfural was quantitatively characterized based on ion *m*/*z* (96.00) and a residence time of 292.5 s. At the same time, the M5HF2C toxin in the reaction system was characterized using SCIEX API4000 LC-MS/MS (AB Sciex, Framingham, MA, USA).

When 5 mL of BL21(DE3) culture reached a similar value of initial OD_600_, a fixed concentration (1 mM) of IPTG was added to induce protein expression for the same duration (12 h). This total protein was used as a catalyst for the conversion of furfural to furoic acid, and the decrease in furfural and the increase in furoic acid were measured at 24 h.

The LC-MS/MS conditions were as follows:

Column: peptide HSS T3 (waters, 2.1 × 100 mm, 2.5 µm); column temperature: 40 °C; mobile phase: A—0.1% FA ACN B—0.1% FA H_2_O.

The elution gradient of the mobile phase is detailed in Table 3.

The parameters for mass spectrometry of the target components were as follows Table 4:

#### 2.2.6. Construction of *Cladh6* Deletion Mutant Strain

A *Cladh6* deletion mutant of *C. lunata* was constructed through *A*. *tumefaciens*-mediated homologous recombination. The *Cladh6* upstream and downstream homology arm amplification primers were designed based on the genome of the *C. lunata* CX-3 strain (Table 5). CX-3 genomic DNA was extracted using Plant Genomic DNA Kit (TIANGEN, DP305, Beijing, China). The DNA of CX-3 strain was used as template to PCR amplify the *Cladh6* upstream and downstream homology arms. The upstream and downstream homology arms were inserted into pCAMBIA1300qh to construct the *Cladh6* deletion vector pCAMBIA1300qh-Δ*Cladh6*.

#### 2.2.7. Determination of Toxin Synthesis Ability of Δ*Cladh6* and Δ*Cladh6*-C Strains

Δ*Cladh6* strain, Δ*Clpks18* strain, Δ*Cladh6* strain + 200 ng/mL furoic acid, Δ*Clpks18* strain + 200 ng/mL furoic acid, Δ*Clpks18* strain + 200 ng/mL 1,3,6,8-tetrahydroxynaphthalene, and CX-3 strain were cultured in Fries 3 medium for 7 days, followed by the detection of M5HF2C toxin production. For the M5HF2C toxin detection method, refer to Section 2.2.3.

## 3. Results

### 3.1. Effects of Xylose on Furoic Acid Production

**Figure 4:**
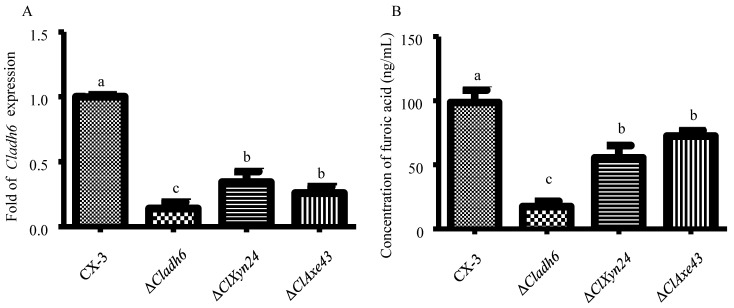
Furoic acid can be sourced from xylose. (**A**) *Cladh6* expression in the Δ*clxyn24*, Δ*claxe43,* and CX-3 strains. (**B**) Concentration of furoic acid in the Δ*clxyn24*, Δ*claxe43*, Δ*Cladh6*, and CX-3 strains. Note: Different letters indicate significant differences at the 0.05 level (*p* < 0.05).

It is established that xylose can be transformed into furfural, which was found to be closely linked to the synthesis of furoic acid in this study. Therefore, we sought to further demonstrate the role of alcohol dehydrogenase in the transformation of xylose to furoic acid. *Cladh6* expression was significantly lower in the Δ*clxyn24* and Δ*claxe43* strain than in the wild-type strain CX-3 (Figure 4A). Therefore, it is hypothesized that M5HF2C toxin originates from xylose through a several-step transformation. This hypothesis is partially confirmed by subsequent experiments. Since furoic acid was found to be significantly lower in the Δ*clxyn24*, Δ*claxe43*, and Δ*Cladh6* strains than in the CX-3 strain (Figure 4B), we supposed that the furoic acid was partially derived from xylose.

### 3.2. Xylose Is Used as a Raw Material for the Biosynthesis of Furoic Acid

**Figure 5:**
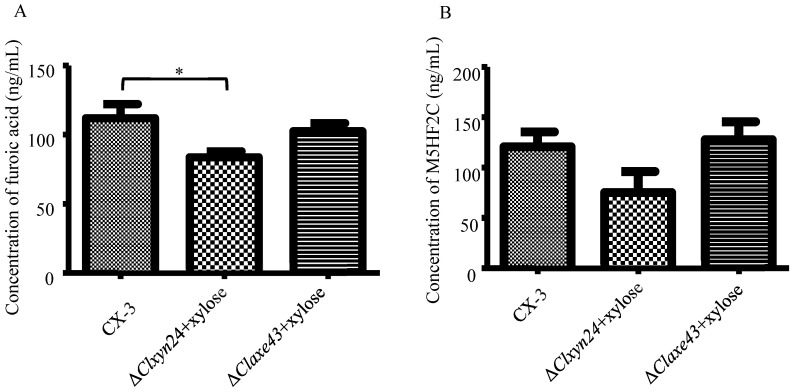
Xylose is one of the precursors in furoic acid biosynthesis. Xylose anaplerosis experiments showing production of (**A**) furoic acid and (**B**) M5HF2C toxin for each strain. Note: The Δ*clxyn24* and Δ*claxe43* strains did not differ significantly from the wild-type strain CX-3 in the levels of M5HF2C toxin after xylose supplementation. Note: Asterisks (*) indicate significant differences at the 0.05 level.

As the results show, once the xylose was added as the sole carbon source to the medium in which the Δ*clxyn24* and Δ*claxe43* strains were cultured, the ability of the Δ*clxyn24* and Δ*claxe43* strains to produce furoic acid and M5HF2C toxin was restored to the level of the wild-type strain CX-3 (Figure 5, Table S1). This confirms that xylose can be used as a raw material for the biosynthesis of furoic acid and affects the biosynthesis of M5HF2C toxin.

### 3.3. Cladh6 Plays an Important Role in M5HF2C Toxin Synthesis

**Figure 6:**
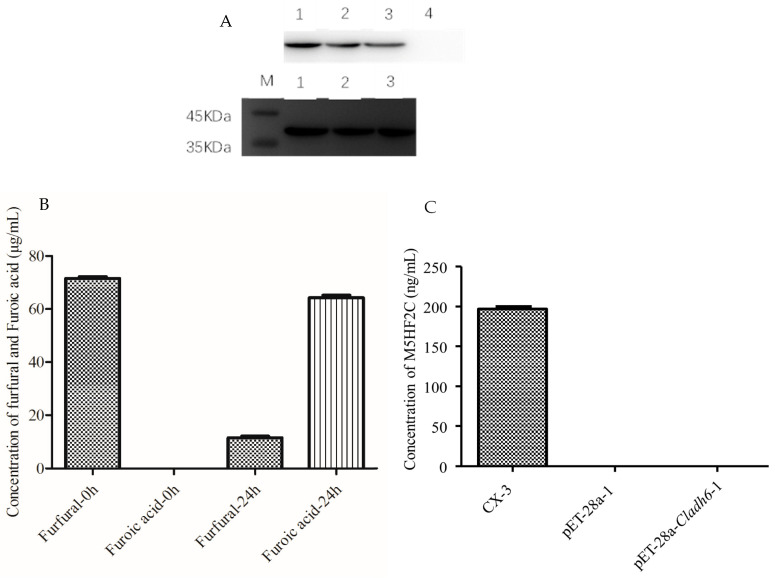
Determination of CLADH6 enzyme activity. (**A**) CLADH6 Western blot and antibody hybridization. Lanes 1–3: (induction of BL21(DE3) strains transferred into pET-32a-Cladh6 for 24 h, 18 h, 12 h); lane 4: (induction of BL21(DE3) strains transferred into pET-32a for 24 h). (**B**) CLADH6 enzyme activity assay in the CLADH6 prokaryotic expression system. (**C**) Detection of M5HF2C toxin in prokaryotic expression systems. Note: CLADH6 possesses the activity for converting furfural to furoic acid, and M5HF2C toxin was not detected in *E. coli* expressing CLADH6.

Through the measurement of CLADH6 enzyme activity, it was demonstrated that CLADH6 is able to catalyze the oxidation of furfural to furoic acid (Figure 6B), but M5HF2C toxin was not detected in the CLADH6 prokaryotic expression system (Figure 6C), indicating that for the oxidation of furfural to furoic acid in the formation of M5HF2C toxin, other enzymes are also required for catalysis.

### 3.4. Demonstration of Furfural Transformed into M5HF2C Toxin in C. lunata

**Figure 7:**
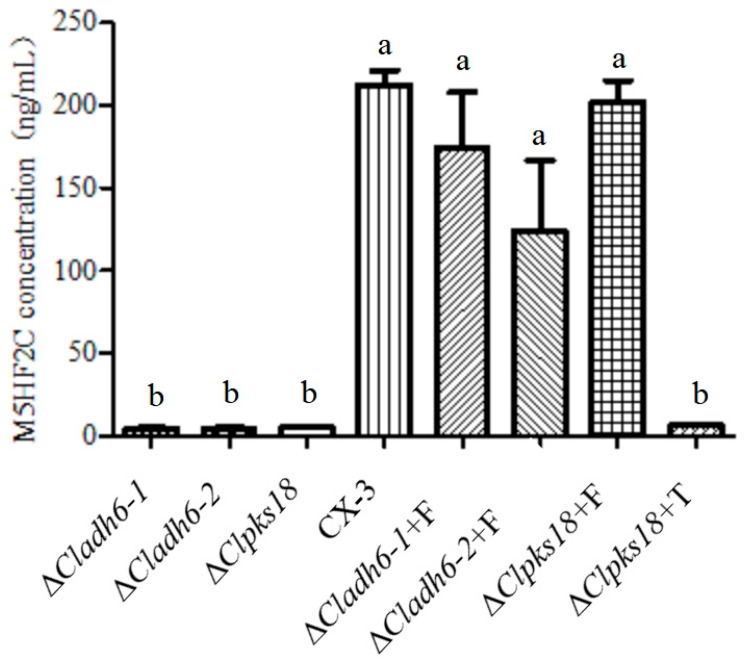
M5HF2C toxin production by CX-3 and *Cladh6* mutant strains and different treatments. Note: +F indicates the addition of 200 ng/mL of furoic acid, and +T indicates the addition of 200 ng/mL of 1,3,6,8-THN. Note: Different letters indicate significant differences at the 0.05 level (*p* < 0.05).

The ability of the Δ*Cladh6* and Δ*Clpks18* strains to produce M5HF2C toxin was significantly reduced compared with the wild-type strain CX-3. However, supplementing the Δ*Clpks18* strain with 1,3,6,8-tetrahydroxynaphthalene (1,3,6,8-THN) at a concentration of 200 ng/mL did not increase its production of M5HF2C toxin. For both the Δ*Cladh6* and Δ*Clpks18* strains, addition of furoic acid at a concentration of 200 ng/mL can increase the production of M5HF2C toxin to the level of the wild-type strain CX-3 (Figure 7). This demonstrates that the M5HF2C toxin is not derived from 1,3,6,8-THN synthesized by the PKS encoded by *Clpks18* and that furoic acid is the precursor of M5HF2C toxin.

### 3.5. Synthesis Pathway from Xylose to M5HF2C Toxin

**Figure 8:**
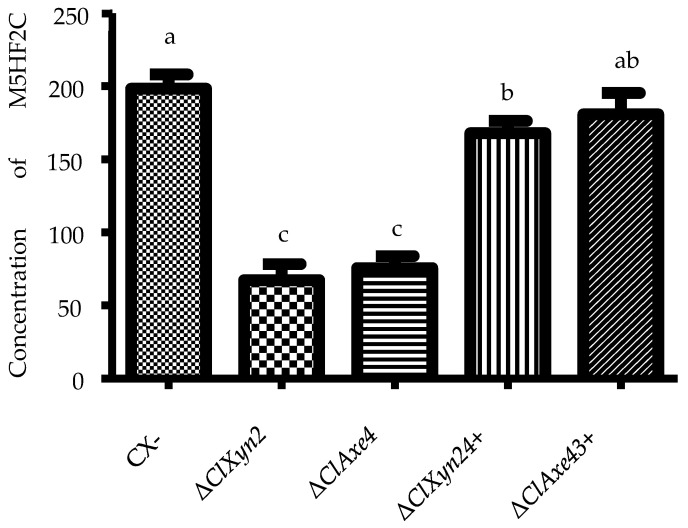
M5HF2C toxin production by the CX-3, Δ*clxyn24,* and Δ*claxe43* strains and different treatments. Note: +F indicates the addition of 200 ng/mL of furoic acid; different letters indicate significant differences at the 0.05 level (*p* < 0.05).

The ability of the Δ*clxyn24* and Δ*claxe43* strains to produce M5HF2C toxin was essentially restored to the level of the wild-type strain CX-3 after the addition of exogenous furoic acid (Figure 8). This suggests that the pathway for biosynthesis of M5HF2C toxin begins with xylose.

## 4. Discussion

In this study, we first identified the basic process for synthesis of M5HF2C toxin in *Curvuaria lunata* following the successive production of precursor or intermediates xylose, furfural, and furoic acid.

The structure of M5HF2C toxin was reported for the first time in a previous study by our group. Meanwhile, a zinc finger protein containing the BTB structural domain was identified to be closely related to the synthesis of M5HF2C toxin, and the encoding gene was termed *Clt-1* [3]. Previous studies by our group have shown that there is significantly lower accumulation of the three intermediates xylose, xylitol, and pyruvic acid in Δ*Clt*-*1*, Δ*ClXyn24*, and Δ*ClAxe43* than in the wild-type strain CX-3. The interaction of *Clt-1*, a protein related to M5HF2C toxin production, with full-length ClXyn24 and ClAxe4 has been confirmed. However, the capabilities of M5HF2C production were increased in all strains when using xylose powder instead of xylan. In addition, Δ*Clt-1*, Δ*ClXyn24*, Δ*ClAxe43,* and Δ*ClXyn24*&*ClAxe43* displayed lower production of acetyl-CoA and malonyl-CoA than WT. Furthermore *Clt-1* was found to physically interact with *ClXyn24* and *ClAxe43* through its BTB domain, which acts upstream of xylose metabolism [4]. The utilization of xylan provides acetyl-CoA and the energy for toxin synthesis, and it was proposed that furfural can be synthesized from xylose via different pathways [8]. Thus, there is a strong case for the fundamental possibility that xylose generated from xylan metabolism is used for the synthesis of furfural in *C*. *lunata*, since xylose is closely linked with furfural formation [5]. In previous studies by our research group, it was speculated that, after xylan is degraded into xylose, xylulose 5-phosphate and xylulone 5-phosphate are formed under the action of xylose reductase, xylitol dehydrogenase, and xylulokinase. Sugar generates acetyl-CoA through pentose phosphate, glycolysis, the tricarboxylic acid cycle, and other pathways. Acetyl-CoA is assembled on PKS18 to form the initial polyketide, and the M5HF2C toxin is then synthesized under the action of a series of enzymes. Xylose is converted into acetyl-CoA via the PPP-EMP pathway, and M5HF2C toxin is then synthesized [4]. It is reported that under catalytic conditions, xylose substances can generate furfural through oxidation under alkaline conditions, and xylan can also generate furfural through hydrolysis [9,10]. Therefore, we hypothesized that under the culture conditions of this experiment furfural originated from xylose.

Earlier studies in *C*. *lunata* have shown that silencing of *Brn1* (trihydroxynaphthalene reductase), a key gene in melanin synthesis, abolishes the ability to produce M5HF2C toxin, suggesting that the *Brn1* gene is involved in the biosynthesis not only of melanin but also of toxins [11]. This suggests that the pathways of toxin and melanin biosynthesis in *C*. *lunata* are intertwined. *Brn1* is one of five key enzymes required for the ab initio synthesis of DHN-melanin in the plant pathogenic fungus [12]. It was found in early studies that *C*. *lunata* is unable to produce melanin when *Brn1* is not expressed, and there is a synergistic relationship between *Brn1* expression and DHN-melanin formation. *Brn1* was expressed in *E*. *coli* from the pGex vector, and the extracted and purified protein was able to convert scytalone to 1,3,8-tri-THN in NADP+, but the activity was relatively low [13].

**Figure 9:**
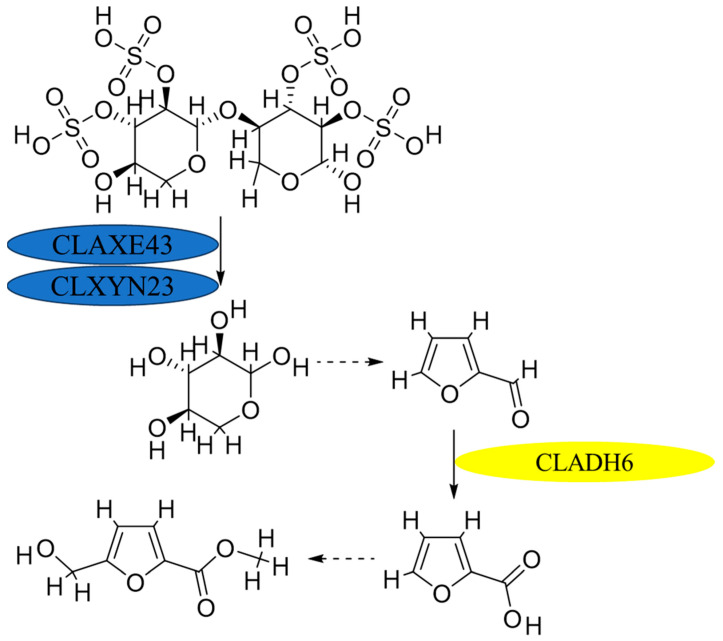
Synthetic pathway from sugars to M5HF2C toxin. Note: Starting point of the M5HF2C synthesis process [4].

In previous research, the *Clpks18*-deletion mutant (Δ*Clpks18*) exhibited mycelial and conidial albinism, suggesting that *Clpks18* is a key gene in melanin synthesis. At the same time, Δ*Clpks18* did not produce M5HF2C toxin, and Δ*Clpks18* demonstrated significantly lower pathogenicity on maize leaves than the wild-type strain (CX-3) [14]. Based on the above studies, previous researchers hypothesized that acetyl coenzyme A is a precursor for M5HF2C toxin synthesis. They have speculated that acetyl coenzyme A is generated from xylose via the PPP-EMP pathway (Figure 1) [4]. However, our recent research showed that the Δ*Clpks18* strain can also produce trace amounts of M5HF2C toxin [6]. Using the SMURF system, 36 secondary metabolic backbone genes were identified from the genome of the CX-3 strain of maize, of which 16 were polyketide synthases (PKSs). Based on the analysis of the structural domains of polyketide synthases and evolutionary analyses, the polyketide synthases were classified according to the presence or absence of a dehydrogenase (DH) domain. Those with DH domains are reductive polyketide synthases, including CLPKS3, CLPKS4, CLPKS6, CLPKS8, CLPKS10, CLPKS11, CLPKS12, CLPKS13, CLPKS14, and CLPKS15, while those without DH domains are nonreducing polyketide synthases, including CLPKS7 and CLPKS9. Because CLPKS9 and *Brn1* are on the same gene cluster and located in close proximity to each other, it was then hypothesized in that study that they catalyze reactions on the same pathway. It has been further hypothesized that they function together in the synthesis of melanin and M5HF2C toxins [15]. Furthermore, the new research shows that CLPKS9 is not involved in the synthesis of either M5HF2C toxin or melanin. CLPKS18 is directly involved in melanin synthesis but not M5HF2C toxin synthesis [6]. PKS1 from *Bipolaris oryzae* is 90.27% homologous (97% coverage) to CLPKS18 from *C*. *lunata* wild-type strain CX-3. Mutating *Bopks1* by T-DNA insertion results in *B*. *oryzae* mycelia albinism [16]. The arrangement of the various functional structural domains of PKSs from *Glarea lozoyensis* and *Nodulisporium* sp. is also similar to that of PKS18, and their products are annotated by antiSMASH as 1,3,6,8-THN [17]. Knockout of the KS and AT regions of the PKS of *Nodulisporium* sp. by homologous recombination results in *Nodulisporium* sp. being unable to produce DHN-melanin [18]. Taking into account the existing literature and experimental results, we conclude that CLPKS18 mainly exerts its function in the DHN-melanin pathway from acetyl-CoA to 1,3,6,8-THN. Therefore, based on the above findings, it was suggested that the precursor of M5HF2C toxin is not acetyl coenzyme A. In summary, we conclude that furfural, as the precursor of M5HF2C toxin, is derived from xylose (Figure 9) but independent of the PPP-EMP pathway and unrelated to acetyl coenzyme A (Figure 1).

According to the previous genomics analysis, there are six ethanol dehydrogenases in the genome of *C*. *lunata*. When the gene *Cladh*6 was deleted, a small amount of the M5HF2C toxin was still detected in the Δ*Cladh*6 strain, and it was speculated that the other alcohol dehydrogenases may also have some activity in converting furfural to furoic acid. It is just that CLADH6 plays a dominant role in the process of M5HF2C toxin biosynthesis.

Nevertheless, further study is warranted regarding whether *Clpks18* affects conversion of xylose into furfural by regulating a certain reaction in the biosynthesis of M5HF2C toxin in *C. lunata*. Taken together, it was confirmed that the biosynthesis process from xylose to furfural, and then to furoic acid, is one of the important steps in the biosynthesis of M5HF2C toxin in *C*. *lunata*.

## 5. Conclusions

Based on the structural analysis of M5HF2C, furfural was first identified as a candidate M5HF2C toxin precursor substance. Through prokaryotic expression analysis, it was demonstrated that the alcohol dehydrogenase CLADH6 in *C. lunata* can catalyze the dehydrogenation of furfural to generate furoic acid, which is an important intermediate in the synthesis of M5HF2C toxin. In this study, we demonstrated that xylose is one of the sources of the furfural and the M5HF2C toxin synthesis pathway is independent of the PPP-EMP pathway and acetyl coenzyme A.

## Figures and Tables

**Table 1:** Elution gradient.

Time (min)	A (%)	B (%)	Flow Rate (mL/min)
0	95	5	0.4
1	95	5	0.4
3	40	60	0.4
4	5	95	0.4
6	5	95	0.4
6.1	95	5	0.4
8	95	5	0.4

**Table 2:** The mass spectrometry parameters.

Precursor Ion	Daughter Ion	Declustering Voltage (V)	Collision Energy (V)
157.0	139.0	70	20
	79.0	70	25

**Table 3:** Elution gradient.

Time (min)	A (%)	B (%)	Flow Rate (mL/min)
0	95	5	0.2
2	95	5	0.2
6	40	60	0.2
8	5	95	0.2
12	5	95	0.2
12.1	95	5	0.2
16	95	5	0.2

**Table 4:** Secondary Mass Spectrometry Information.

Q1 Mass (Da)	Q3 Mass (Da)	Time (msec)	ID	DP (volts)	CE (volts)
		negative ion			
154.8	109.9	100	ME	−42.09	−42.09

**Table 5:** Primers for *Cladh6* deletion mutations and backfill strains.

Primer Name	Sequence
Cladh6Uf	ACGACGGCCAGTGCCAAGCTTTCTGGAAGACGACCCTGGCGA
Cladh6Ur	GACCTGCAGGCATGCATTTGGCGGGTACAGATGTTG
1300qh-F	GCATGCCTGCAGGTCGACTCT
Cladh6D + 1300qh-RB	GTGAATTAGTGTACGGAGCTCGGTACCCGGGGATC
Cladh6Df	CGTACACTAATTCACATATACGGCT
Cladh6Dr	TATGACCATGATTACGAATTCCTAGCCTAAGGTTGGTGAAGG
HygR1	ACCGCAAGGAATCGGTCAAT
HygR2	GATTTGTGTACGCCCGACAG

## Data Availability

The original contributions presented in the study are included in the article and Supplementary Materials, further inquiries can be directed to the corresponding author.

## Supplementary Materials

The following supporting information can be downloaded at: https://www.mdpi.com/article/10.3390/jof10100688/s1, Figure S1: Standard Curve and Peak Area Integration of Figure 2; Figure S2. Standard Curve and Peak Area Integration of Figure 2A; Figure S3 Standard Curve and Peak Area Integration of Figure 2B; Figure S4 Prokaryotic expression of *Cladh6*; Figure S5 GC-MS spectrum of furfural standard; Figure S6 GC-MS spectrum of furoic acid standard; Table S1: The list of raw data from xylose supplementation experiments.
