# Supplementary material for: Elucidating the Fundamental Process of Methyl-(5hydroxymethyl) Furan-2-Carboxylate Toxin Biosynthesis in Curvularia lunata Causing Maize Leaf Spot
Source: J Fungi (Basel). 2024 Sep 30;10(10):688. doi: 10.3390/jof10100688 (PMC11508280; doi:10.3390/jof10100688)
Supplement: Supplementary file 1 [file jof-10-00688-s001.zip › jof-3021010-supplementary.pdf]

Supplementary Materials

Regression Equation: y = 136 x + 1.08e+003 (r = 0.9999)

Expected Concentration	Number of Values	Mean Calculated Concentration	% Accuracy
4.49	1	5.03	112.0
8.98	1	9.17	102.2
18	1	16.34	90.8
35.9	1	36.91	102.8
144	1	143.68	99.8

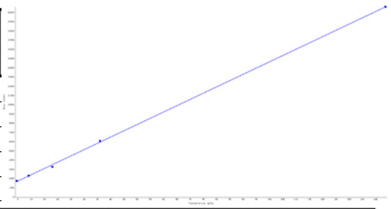

CX3-1

RT (Exp. RT): 1.59 (1.59) min

[Calculated Conc]: 99.1 ng/mL

Area: 1.45e+004

Sample Type: (Unknown)

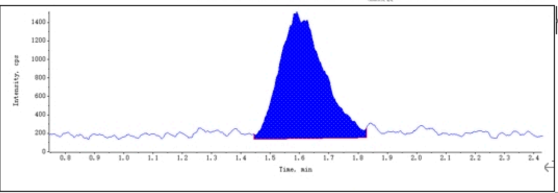

CX3-2

RT (Exp. RT): 1.81 (1.59) min

[Calculated Conc]: 108. ng/mL

Area: 1.57e+004

Sample Type: (Unknown)

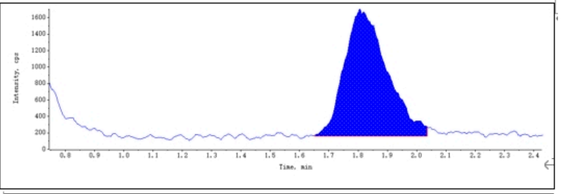

CX-3-1a

RT (Exp. RT): 1.59 (1.59) min

[Calculated Conc]: 88.5 ng/mL

Area: 1.31e+004

Sample Type: (Unknown)

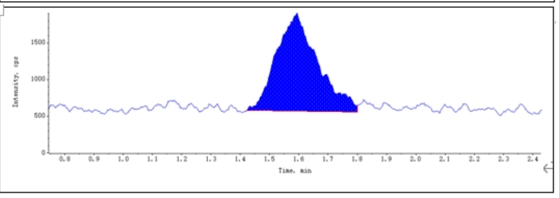

△cladh6-1

RT (Exp. RT): 1.84 (1.59) min

[Calculated Conc]: 12.6 ng/mL

Area: 2.79e+003

Sample Type: (Unknown)

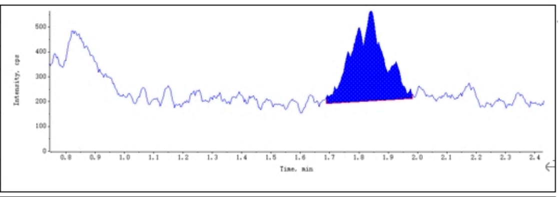

△cladh6-2

RT (Exp. RT): 1.84 (1.59) min

[Calculated Conc]: 19.9 ng/mL

Area: 3.79e+003

Sample Type: (Unknown)

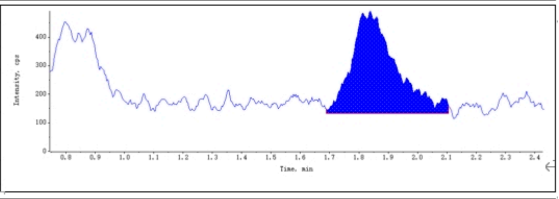

△cladh6-3

RT (Exp. RT): 1.59 (1.59) min

[Calculated Conc]: 19.7 ng/mL

Area: 3.76e+003

Sample Type: (Unknown)

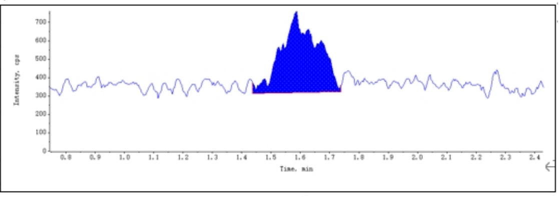

△clXyn24-1

RT (Exp. RT): 1.64 (1.59) min

[Calculated Conc]: 63.8 ng/mL

Area: 9.76e+003

Sample Type: (Unknown)

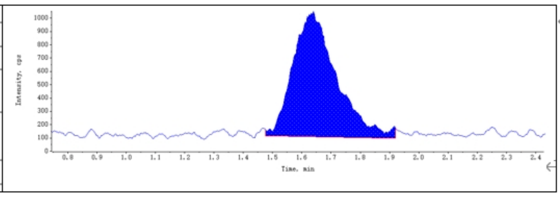

Figure S1 Go to next page

Figure S1 continued from previous page

△clXyn24-2

RT (Exp. RT): 1.83 (1.59) min

[Calculated Conc]: 45.0 ng/mL

Area: 7.20e+003

Sample Type: (Unknown)

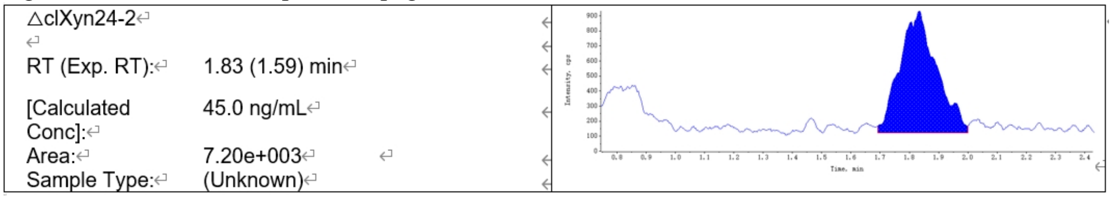

△clXyn24-3

RT (Exp. RT): 1.85 (1.59) min

[Calculated Conc]: 57.5 ng/mL

Area: 8.90e+003

Sample Type: (Unknown)

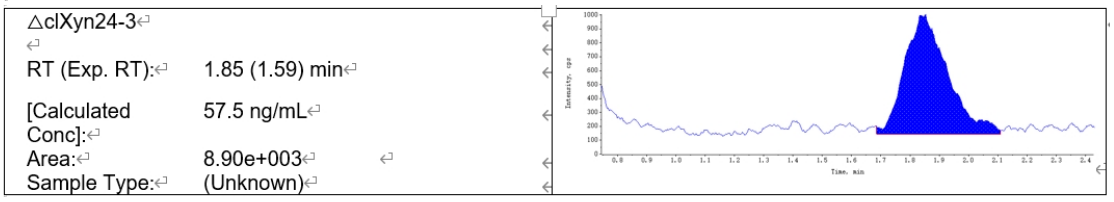

△clAxe43-1

RT (Exp. RT): 1.85 (1.59) min

[Calculated Conc]: 67.5 ng/mL

Area: 1.03e+004

Sample Type: (Unknown)

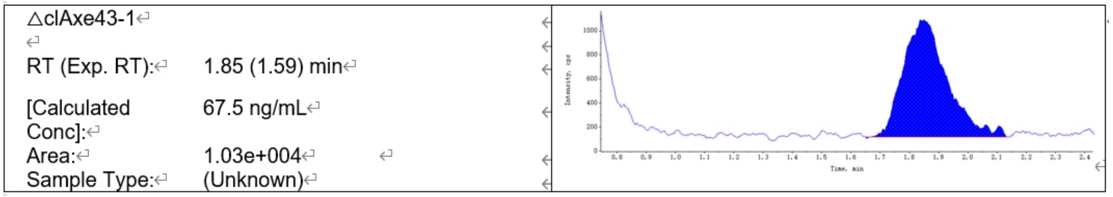

△clAxe43-2

RT (Exp. RT): 1.85 (1.59) min

[Calculated Conc]: 75.7 ng/mL

Area: 1.14e+004

Sample Type: (Unknown)

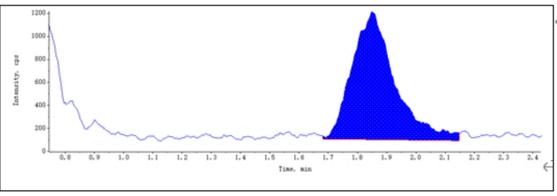

△clAxe43-3

RT (Exp. RT): 1.84 (1.59) min

[Calculated Conc]: 74.1 ng/mL

Area: 1.12e+004

Sample Type: (Unknown)

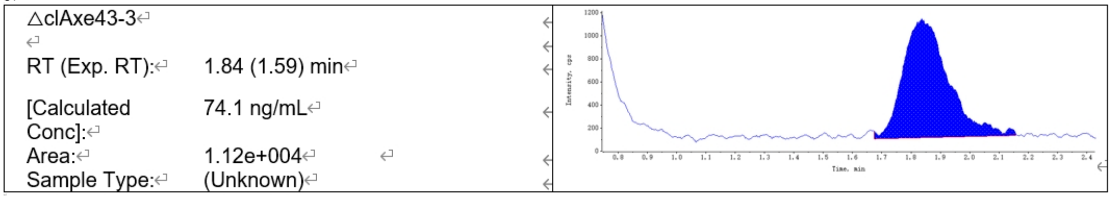

Figure S1. Standard Curve and Peak Area Integration of Figure 2-1

Regression Equation: y = 320 x + 3.39e+003 (r = 0.9999)

Expected Concentration	Number of Values	Mean Calculated Concentration	% Accuracy
19.5	1	13.97	71.7
39.1	1	40.01	102.3
78.1	1	77.62	99.4
156	1	162.78	104.3
313	1	312.58	99.9
625	1	623.50	99.8

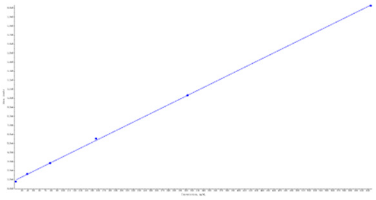

Analyte: FA (110.900/67.000 Da)

CX-3-1

RT (Exp. RT): 1.74 (1.75) min

[Calculated Conc]: 506.ng/mL

Area: 1.65e+005

Sample Type: (Unknown)

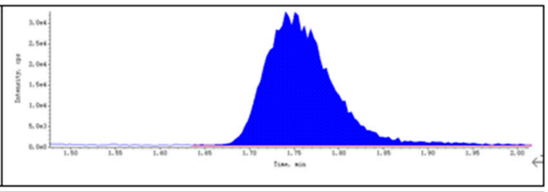

CX-3-2

RT (Exp. RT): 1.74 (1.75) min

[Calculated Conc]: 608.ng/mL

Area: 1.98e+005

Sample Type: (Unknown)

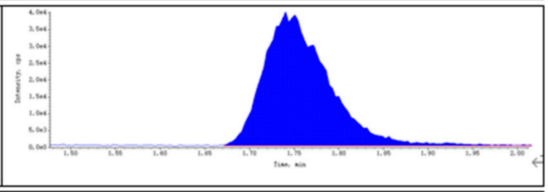

CX-3-3

RT (Exp. RT): 1.74 (1.75) min

[Calculated Conc]: 565.ng/mL

Area: 1.84e+005

Sample Type: (Unknown)

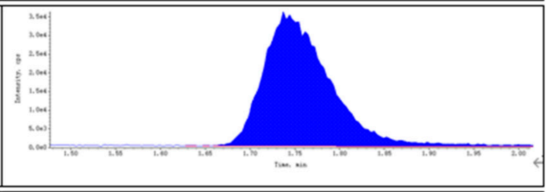

△clxyn24-1

RT (Exp. RT): 1.74 (1.75) min

[Calculated Conc]: 403.ng/mL

Area: 1.32e+005

Sample Type: (Unknown)

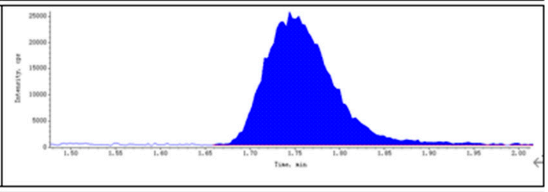

△clxyn24-2

RT (Exp. RT): 1.75 (1.75) min

[Calculated Conc]: 434.ng/mL

Area: 1.42e+005

Sample Type: (Unknown)

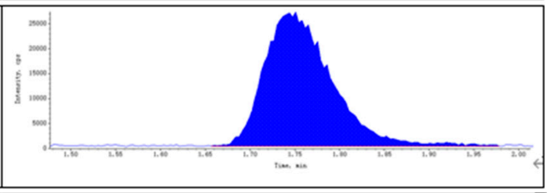

△claxe43-1

RT (Exp. RT): 1.75 (1.75) min

[Calculated Conc]: 533.ng/mL

Area: 1.74e+005

Sample Type: (Unknown)

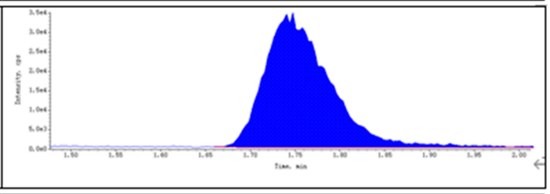

△claxe43-3

RT (Exp. RT): 1.75 (1.75) min

[Calculated Conc]: 493.ng/mL

Area: 1.61e+005

Sample Type: (Unknown)

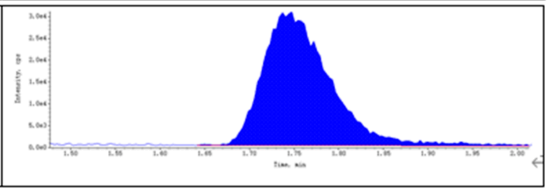

Figure S2. Standard Curve and Peak Area Integration of Figure 2-2 A

The Δ*clxyn24* and Δ*claxe43* are both with xylose supplementation

Regression Equation: y = 5.91e+003 x + 2.17e+004 (r = 0.9995)

Expected Concentration	Number of Values	Mean Calculated Concentration	% Accuracy
3.68	1	0.02	0.5
7.37	1	4.39	59.6
14.7	1	12.59	85.7
29.5	1	27.41	92.9
58.9	1	62.01	105.3
118	1	121.43	102.9
236	1	246.18	104.3
472	1	465.28	98.6

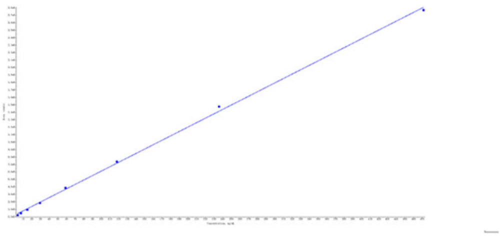

Analyte: M5H2F **(**157.600/139.000 Da**)**

CX-3-1

RT (Exp. RT): 3.53 (3.53) min
[Calculated Conc]: 260.ng/mL
Area: 1.56e+006
Sample Type: (Unknown)

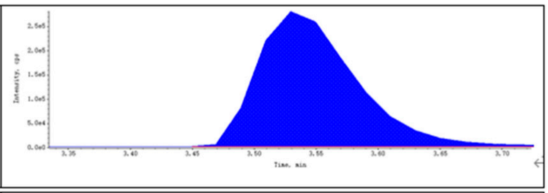

CX-3-2

RT (Exp. RT): 3.53 (3.53) min
[Calculated Conc]: 316.ng/mL
Area: 1.89e+006
Sample Type: (Unknown)

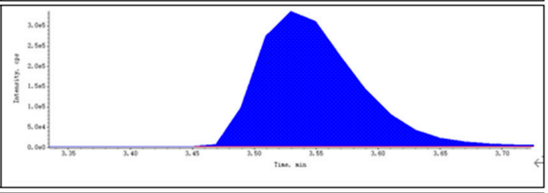

CX-3-3

RT (Exp. RT): 3.53 (3.53) min
[Calculated Conc]: 330.ng/mL
Area: 1.97e+006
Sample Type: (Unknown)

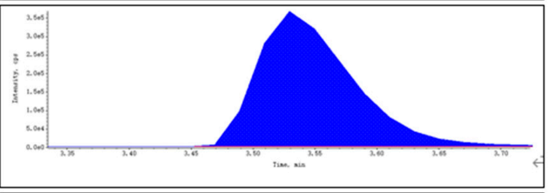

△clxyn24-1

RT (Exp. RT): 3.53 (3.53) min
[Calculated Conc]: 151.ng/mL
Area: 9.14e+005
Sample Type: (Unknown)

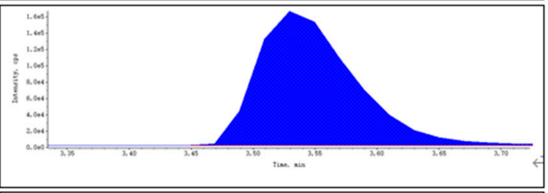

△clxyn24-2

RT (Exp. RT): 3.53 (3.53) min
[Calculated Conc]: 225.ng/mL
Area: 1.35e+006
Sample Type: (Unknown)

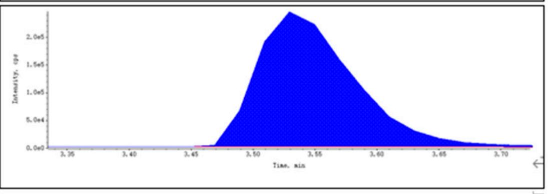

△claxe43-1

RT (Exp. RT): 3.53 (3.53) min
[Calculated Conc]: 351.ng/mL
Area: 2.10e+006
Sample Type: (Unknown)

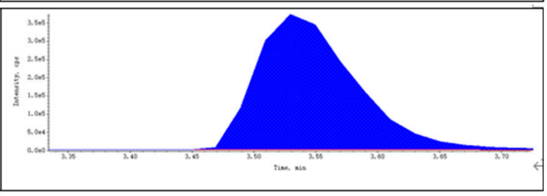

△claxe43-3

RT (Exp. RT): 1.75 (1.75) min
[Calculated Conc]: 493.ng/mL
Area: 1.61e+005
Sample Type: (Unknown)

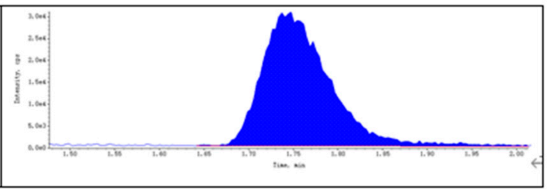

Figure S3. Standard Curve and Peak Area Integration of Figure 2-2 B

The Δ*clxyn24* and Δ*claxe43* are both with xylose supplementation

Table S1. The list of raw data from xylose supplementation experiments

Sample No.	Concentration of furoic acid (ng/mL)	Concentration of M5HF2C (ng/mL)
CX-3-1	101.20	104.00
CX-3-2	121.60	126.40
CX-3-3	113.00	132.00
△clxyn24-1	80.60	60.40
△clxyn24-2	86.80	90.00
△claxe43-1	106.60	140.40
△claxe43-3	98.60	115.20

The Δ*clxyn24* and Δ*claxe43* are both with xylose supplementation

Note for **Figure S2**, **Figure S3** and **Table S1**: The samples are concentrated 5-fold for the determination of FA concentration and 2.5-fold for the determination of M5HF2C concentration by UHPLC-MS. The data in Table S1 are converted. Due to the exclusion of 2 sets of abnormal data, CX-3 was not paired with other mutants supplementation with xylose in the treatment group data. Therefore, differences between groups were compared by a two-tailed t test.

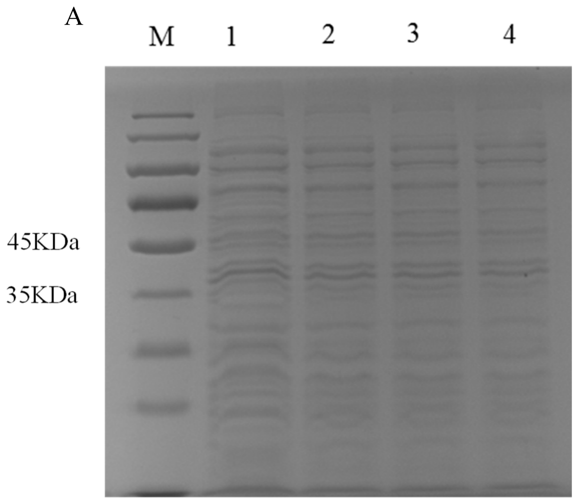

Figure S4. Prokaryotic expression of *Cladh6*

SDS-PAGE electrophoresis of proteins induced by BL21(DE3) strains expressing pET-32a-Cladh6. 1-3: induction of BL21(DE3) strains transferred into pET-32a-Cladh6 for 24 h, 18 h, 12 h, 4: induction of BL21(DE3) strains transferred into pET-32a for 24 h.

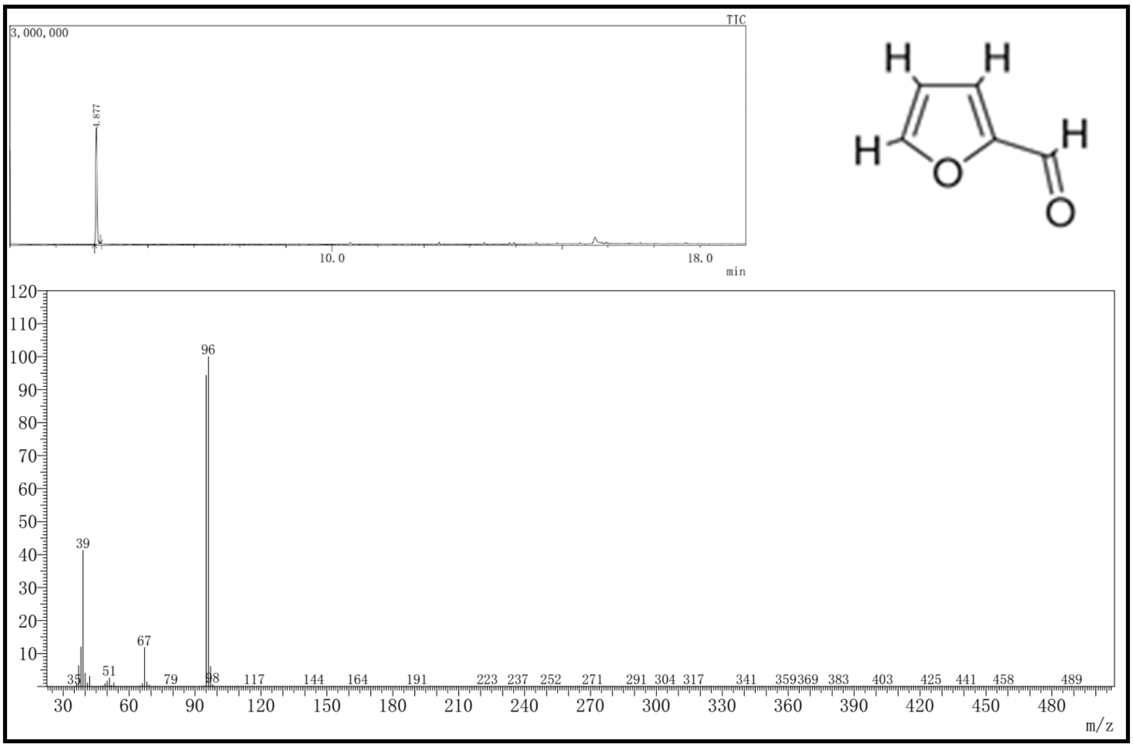

Figure S5. GC-MS spectrum of furfural standard

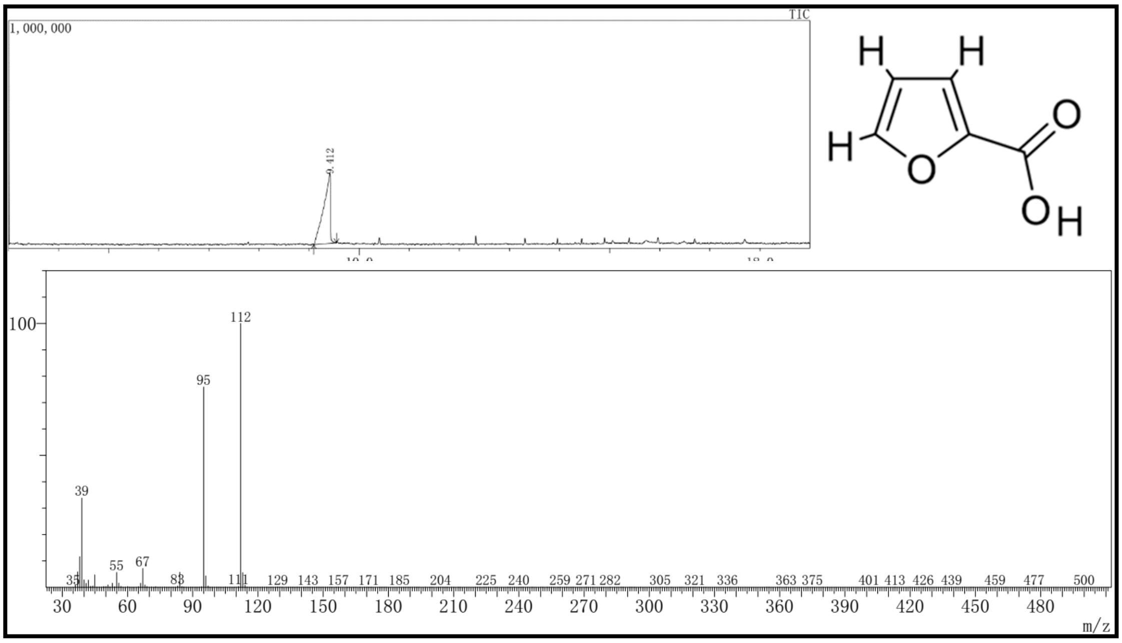

Figure S6. GC-MS spectrum of furoic acid standard
